# Unfolding Novel Mechanisms of Polyphenol Flavonoids for Better Glycaemic Control: Targeting Pancreatic Islet Amyloid Polypeptide (IAPP)

**DOI:** 10.3390/nu9070788

**Published:** 2017-07-21

**Authors:** Ivana R. Sequeira, Sally D. Poppitt

**Affiliations:** 1 Human Nutrition Unit, School of Biological Sciences, University of Auckland, Auckland 1010, New Zealand; i.sequeira@auckland.ac.nz; 2 High-Value Nutrition National Science Challenge, Auckland 1142, New Zealand; 3 Department of Medicine, University of Auckland, Auckland 1142, New Zealand

**Keywords:** type 2 diabetes, insulin resistance, β-cell dysfunction, polyphenols, flavonoids, protein misfolding disease (PMD), rutin, quercetin-*O*-rutinoside, antioxidant, islet amyloid polypeptide (IAPP), amyloidogenesis

## Abstract

Type 2 diabetes (T2D) is characterised by hyperglycaemia resulting from defective insulin secretion, insulin resistance, or both. The impact of over-nutrition and reduced physical activity, evidenced by the exponential rise in obesity and the prevalence of T2D, strongly supports the implementation of lifestyle modification programs. Accordingly, an increased consumption of fruits and plant-derived foods has been advocated, as their intake is inversely correlated with T2D prevalence; this has been attributed, in part, to their contained polyphenolic compounds. Over the last decade, a body of work has focussed on establishing the mechanisms by which polyphenolic compounds exert beneficial effects to limit carbohydrate digestion, enhance insulin-mediated glucose uptake, down-regulate hepatic gluconeogenesis and decrease oxidative stress; the latter anti-oxidative property being the most documented. Novel effects on the inhibition of glucocorticoid action and the suppression of amylin misfolding and aggregation have been identified more recently. Amyloid fibrils form from spontaneously misfolded amylin, depositing in islet cells to elicit apoptosis, beta cell degeneration and decrease insulin secretion, with amyloidosis affecting up to 80% of pancreatic islet cells in T2D. Therefore, intervening with polyphenolic compounds offers a novel approach to suppressing risk or progression to T2D. This review gives an update on the emerging mechanisms related to dietary polyphenol intake for the maintenance of glycaemic control and the prevention of T2D.

## 1. Introduction

Worldwide, the number of people diagnosed with diabetes mellitus (DM) has more than doubled over the past three decades [1], with an estimated 285 million individuals in 2010 [2], of which 90% had type 2 diabetes (T2D) [3]. The main pathophysiologic drivers of T2D are insulin resistance (IR) and pancreatic β-cell dysfunction. IR occurs when the body becomes less sensitive or becomes resistant to the action of insulin, manifesting as inadequate insulin-mediated suppression of hepatic gluconeogenesis and inadequate glucose disposal from circulation [4]. Pancreatic β-cells, in turn, are the insulin and amylin-secreting cells of the pancreas, which may alter in both structure and function during a disease state. Each presents at distinct times during the course of disease progression, with IR proposed to be the primary driver and β-cell dysfunction a later manifestation [5]. The etiology of these factors is distinct. IR may result from a defect in insulin signalling, a defect in glucose transporters or lipotoxicity. Conversely, β-cell dysfunction is postulated to be caused by amyloid deposits in the pancreatic islet’s cells, oxidative stress and increased fatty acids within the pancreas, or limited incretin action [6]. Recent studies provide data to suggest that the accumulation of islet amyloid polypeptide (IAPP) or amylin, which is co-secreted with insulin in the pancreatic β-cells, worsens pancreatic function, fast-tracking progression to T2D [7]. Emerging evidence demonstrates that it is the gradual accumulation of the IAPP fibrils, rather than the amyloid deposit itself, that is cytotoxic [8], resulting in increased oxidative stress and membrane permeability [9,10]; both features critical to the pathogenesis of T2D [11]. Accordingly, the role of polyphenol flavonoids is receiving particular interest, given that they have been shown to interfere with the amyloid assembly pathway to inhibit the formation of amylin aggregates, associated cytotoxicity and pancreatic β-cell apoptosis [12,13]. The favourable molecular structure of flavonoids enables them to chemically bind to and prevent assembly of the IAPP fibrils and is now emerging as a possible therapeutic strategy for preventing and delaying progression to T2D [14]. IR most often precedes the onset of T2D and is compensated for by the increased secretion of insulin from islet β-cells to maintain normal circulating glucose levels. This was established in early studies by Reaven and colleagues [15,16,17] which demonstrated that approximately 25% of nondiabetic individuals exhibit IR within the range of that observed in T2D patients. A deterioration into an inability to regulate blood glucose, both when fasted and following a meal, occurs when either IR increases or β-cell insulin secretory responses decrease, or both.

The alarming increase in the prevalence of T2D, once considered a health issue that plagued Western industrialised nations, has led to significant concern in developing countries [18,19]. On the basis of population growth rates, the aging generation and rates of urbanisation, it has been estimated that two Asian countries—India and China—will shoulder the global T2D burden by the year 2030 [2]. Asian ethnicities appear to be more susceptible to T2D than their Caucasian counterparts, despite commonly being of lower body weight and body mass index (BMI). This may be caused in part by ectopic lipid infiltration into key metabolic organs such as the pancreas and liver [20], the underpinning mechanism for which is as yet not determined but is purported to be due in part to a consequence of lipid ‘overspill’ from peripheral or central adipose depots during weight gain. One of the key factors attributed to the growth of the diabesity epidemic is a marked change in lifestyle and dietary habits as a result of environmental changes and globalisation [21,22]. Evidence from epidemiological studies indicate that 90% of cases of incident T2D can be attributed to being overweight or obese, over-nutrition, lack of physical activity, smoking, and alcohol consumption [23,24,25]. Hence, ongoing strategies for the management of T2D emphasise the importance of timely intervention through modifiable risk factors, such as dietary and lifestyle changes [26], which are pertinent to not only delaying progression but also preventing the risk of developing T2D [27]. Robust data from several major diabetes prevention trials [26,28,29,30,31] unequivocally show that intensive lifestyle interventions, aimed at weight loss and increased physical activity in high-risk individuals, can prevent or at least delay the progression to overt T2D by 50% [32]. Consequently, considered as effective as intervening with pharmacological agents [33], lifestyle changes are also increasingly promoted as cost-effective [34], affording a maximal benefit with minimal harm [35] to at-risk individuals with poor metabolic health.

The aim of this review is to provide an update on the established mechanisms by which polyphenol flavonoids are known to reduce the risk of T2D and to present a novel mechanism for the inhibition of amylin misfolding and aggregation.

## 2. Polyphenol Flavonoids Are Essential Non-Nutrient Bioactive Molecules, Having Established Mechanisms in Reducing the Risk of T2D

Several studies highlight the benefit of a diet characterised by not only greater quantities but also a greater variety of fruit and vegetables as an important predictor of glucose tolerance and decreased T2D risk [36,37,38,39]. While the precise mechanism by which fruit and vegetables exert their beneficial effects are unknown, the ubiquitous polyphenolic phytochemicals contained within them have been proposed to have favourable effects. Dietary polyphenols constitute approximately 500 compounds with diverse structure and distribution in foods [40]. As they occur widely in plant-derived foods and beverages, it is estimated that the average intake of polyphenolic compounds in the US population is ~1 g/day [41], between ~0.5–2 g/day in European countries [42], and 0.8–1.1 g/day in the UK [43,44]. Common items in a diet enriched with these non-nutrient bioactive polyphenolic compounds include broccoli, onion, cabbage, grapes, apples, cherries, pears, strawberries, oranges, legumes such as soyabean, cocoa and chocolate [45].

Various epidemiological studies further support the beneficial effects of polyphenol-rich diets in preventing and managing T2D [46]. It was reported in the Nurses’ Health Study (*N* = 1111) (NHS I and II) that markers of flavonones (naringenin and hesperetin) and flavonols (quercetin and isorhamnetin) were significantly associated with a 30–48% lower T2D risk during the follow-up period (≤4.6 years (median)) [47]. Total flavonoid (HR: 0.90; 95% CI 0.77–1.04, *p* = 0.04) and flavonol (HR: 0.81; 95% CI 0.69–0.95, *p* = 0.02) intakes were also shown to reduce the risk of T2D in the European prospective investigation into cancer and Nutrition-InterAct (EPIC-InterACT) study [48]. Similarly, higher intakes of anthocyanins and anthocyanin-rich foods were shown to be associated with a significantly lower risk of T2D (pooled HR for 3 cohorts: 0.85 95% CI 0.80–0.91, *p* < 0.001) in the NHSI (*N* = 70,359 women), NHSII (*N* = 89,201 women) and Health Professionals Follow-Up studies (*N* = 41,334 men) [49]. Whole fruit consumption, such as apples [49,50,51], pears [49] and blueberries [49,51] which contain polyphenolic compounds are also reported to be inversely associated with T2D. The beneficial effects of total flavonoid intake or any of the flavonoid subclasses are, however, not observed in some studies [50,52]; this may be due to differences in intakes, variability in absorption following dietary intake [53], the structure of the polyphenol itself and the nature of the food source [54]. Notably, these studies [50,52] utilised self-reported semi-quantitative food frequency questionnaires (FFQs)—a tool commonly used to assess dietary intake—that are recognised to be limited in accuracy, as polyphenol intakes may likely be over or under-estimated by this methodology [55].

A large body of evidence links the antioxidant activity of polyphenols [47,49,56,57,58,59,60] as the primary mechanism by which they lower T2D risk. This is in line with results from a recent meta-analysis [61] which confirmed that the consumption of dietary flavonoids was associated with both the maintenance of body weight and a decreased risk of T2D [59]. The beneficial effects of polyphenols in T2D has also been recently reviewed to expand the effects to include improved carbohydrate metabolism via the modulation of metabolic enzymes and nuclear receptors [62], the alteration of gene expression and signalling pathways [63,64], a reduction in the absorption of simple sugars via the inhibition of α-amylase and α-glucosidase, and also an improved uptake of glucose by muscle and adipocytes [46,64]. Furthermore, polyphenol flavonoids also modulate the release of glucose monomers from glycogen deposits by inhibiting glycogen phosphorylase (GP) to prevent hyperglycaemic episodes [65]. Recently, polyphenol-rich olive leaf extracts (OLE) [66,67,68,69,70] have been shown to improve lipid and glycaemic control in T2D, in line with a recent meta-analysis [71] of 36 controlled, randomised trials using polyphenol-extracts, supplements and foods, ranging from 28 mg to 1.5 g for 0.7–12 months, that showed that polyphenol intake lowered Hb_A1c_ levels by 2.29 ± 0.4 mmol/mol in T2D (*N* = 1426, baseline Hb_A1c_ = 58 mmol/mol).

## 3. Unravelling Novel Mechanisms by Which Polyphenol Flavonoids Further Ameliorate T2D Risk

**Figure 1:**
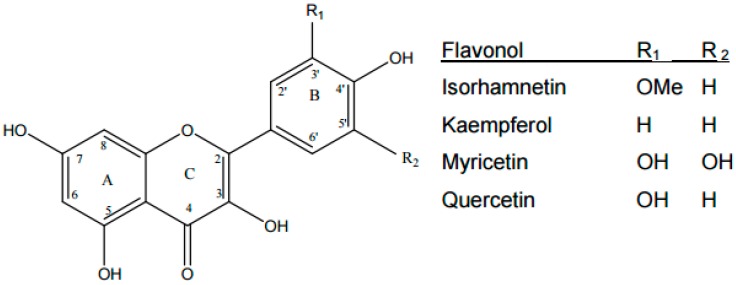
Chemical structure of flavonols: quercetin is the most abundant in the diet and is found in onions, broccoli, apples, tea and red wine.

A novel and important target for the use of dietary polyphenol flavonoids in the prevention of T2D is the misfolding of pancreatic amylin and the subsequent deposition of these aggregates in islet β-cells. Indeed, it is their unique aromatic features and their highly conjugated system with multiple hydroxyl groups that make them ideal candidates for targeting amyloid deposits and, additionally, as effective electron and hydrogen atom donors [63] to neutralise free radicals and other reactive oxygen species (ROS) [72]. All polyphenolic compounds share a common phenolic ring structure, with one or more phenolic rings linked to more than one hydroxyl group [73], and are categorised into three main sub-groups: phenolic acids, flavonoids and non-flavonoids. The flavonoids are the largest class of phenolic compounds and include sub-classes, 6 of which are highlighted according to their nutritional relevance: flavonols, isoflavones, anthocyanidins, flavan-3-ols, flavones and flavanones [53]. Dietary quercetin is the most abundant flavonol and is found in onions, apples, tea, broccoli, and red wine (Table 1) along with kaemferol, isorhamnetin, and myricetin, and is a potent antioxidant [74] (Figure 1).

**Figure 2:**
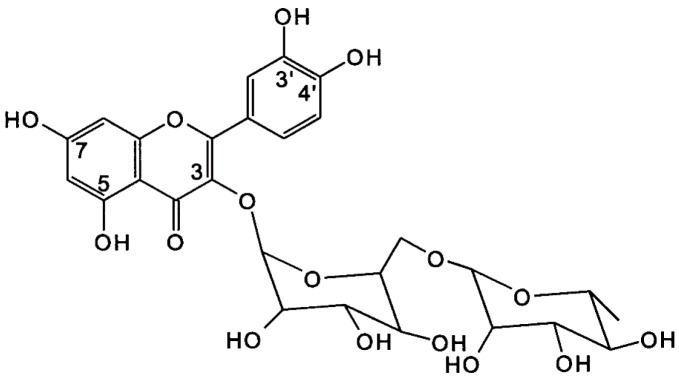
Structure of quercetin-3-*O*-rutinoside (rutin): quercetin commonly occurs conjugated with a sugar moiety at the 5, 7, 3′, 4′ or 5′ position.

Quercetin commonly occurs conjugated with a sugar moiety at the 5, 7, 3′, 4′, or 5′ positions, which is frequently a rutinoside conjugate (rutin) [81], such that the glycoside combines the flavonol quercetin and the disaccharide rutinose. Rutin is thought to be the main glycosidic form of quercetin in the diet [82] and is found abundantly in buckwheat [83,84] (Table 1). Additionally, quercetin has been shown to have a greater antioxidant activity compared to polyphenolic acids without this structure, due to 3-hydroxy groups and conjugated π bonds [73] (Figure 2); hence the purported beneficial effects of quercetin and quercetin-*O*-rutinoside (rutin) in the improvement of glycaemic indices in recent in vitro [85] and animal models [86,87,88,89,90] studies, as well as in preventing amylin misfolding and aggregation [91,92,93,94].

### 3.1. Pancreatic β-Cell Dysfunction Due to Amylin Misfolding and Aggregation

Accumulating evidence suggests that toxic aggregates of islet amyloid polypeptide (IAPP), commonly known as amylin [95], may make a significant contribution to β-cell dysfunction and T2D [8,96,97,98], it is classified as a protein misfolding disease (PMD). PMDs are a common occurrence in conditions when at least one protein or peptide misfolds, aggregates, and accumulates in tissues where damage occurs. This has also been implicated in the pathogenesis of several neurodegenerative disorders, including Alzheimer’s (AD), Parkinson’s (PD) and Huntington disease (HD) [99]. Evidence linking protein misfolding and aggregation with disease comes from post-mortem histopathological studies, where a typical feature of each disease is the accumulation of protein deposits; namely, amyloid β (Aβ) in AD, α-synuclein in PD, and poly-Q expanded Huntington in HD [99]. IAPP is a 4 kDa peptide hormone composed of 37-amino acids that is synthesised and co-secreted along with insulin from pancreatic islet β-cells [100]. IAPP has important glucomodulatory effects, as it slows gastric emptying to regulate glucose levels and inhibits the effect of insulin and arginine-stimulated glucagon release by pancreatic α-cells [101]. Moreover, IAPP is involved in appetite regulation via the gut-brain axis and functions as a growth factor in maintaining β-cell mass [102]. The IAPP monomer is shown to have normal biological activity in healthy islet β-cells, wherein oligomers can form and undergo degradation. It is unclear why this process is dysregulated in IR and T2D [103] to the extent that innate physiochemical properties predispose it to aggregate and form fibrils [8], but growing evidence supports this as a key causative driver of T2D. Notably, glucocorticoid (GC) may have a role in pathology [104], much like that observed in AD to form amyloid β [105]. A GC-mediated increase in β-cell IAPP to insulin secretion has been established following dexamethasone treatment in animals [106,107,108] as well as humans [109]. A major determinant of GC action is the enzymes that convert low active cortisone into active cortisol, especially 11β-hydroxysteroid degydrogenase type 1 (HSD1), mainly expressed in liver and adipose tissue [110], a key mediator of IR [111,112,113] and pancreatic β-cell dysfunction [114].

The association of IAPP accumulation with T2D was first described as ‘islet hyalinosis’ by Eugene Opie [115] and reported to be a common occurrence in 90% of T2D individuals [116,117,118]. Notably, some diabetic patients were reported not to present with islet amyloid [116,119], an important observation which has more recently been explained by the identification of soluble oligomers and aggregates of amylin, which are more cytotoxic than the mature fibrils. Conversely, some normoglycaemic individuals may exhibit these features in line with AD and PD, where disease-free older individuals may also develop protein aggregates [120,121]. Rodent studies show the formation of IAPP aggregates precedes β-cell dysfunction and the clinical signs of disease [122,123,124], suggesting that hyperglycaemia may not be a prerequisite for islet amyloid formation. This has more recently been verified by careful phenotyping of human amylin (hA) transgenic mice, which showed that hA oligomers did not arise as a result of T2D, but were causative of the dysglycaemia [8]. While evidence from clinical post-mortem studies links IAPP aggregation with a loss of β-cell mass and frank T2D [118,125], it is unlikely that large amyloid deposits are required for β-cell degeneration; instead, evidence points to small soluble oligomers as the likely cytotoxic forms of hA. Studies showing misfolded fibrillogenic hA to be cytotoxic and causative of pancreatic islet β-cell apoptosis have identified several cell-signalling pathways, including the activation of Fas-associated death receptor signalling [126] confirmed by Fas blocking antibodies, which suppressed hA-evoked apoptosis.

### 3.2. Mechanisms That Underpin the Formation of Islet Amyloid Aggregates

Amyloidogenesis occurs in three stages, whereby initially the protein misfolds (lag phase) and is rearranged to form the β-sheet (growth phase), which matures to form stable fibrils (saturation phase) or amyloid deposits [14]. Briefly, a model has been proposed for islet amyloid formation [97]. In a healthy state, IAPP is predominantly expressed by pancreatic β-cells as the 89-amino acid, pre-pro-IAPP, which in the endoplasmic reticulum (ER) is cleaved to pro-IAPP and further processed in the Golgi apparatus and secretory granules in a pH-dependent manner. It is noteworthy that cleavage occurs via similar enzymes which process pro-insulin. Processed IAPP is stored with insulin in the secretory granules in a molar ratio of 1-2:50. Insulin and pro-insulin have an inhibitory effect on IAPP aggregation and, together with the low pH within the secretory granules, maintain IAPP in the soluble state. The resultant IAPP is co-secreted along with insulin by the β-cells in response to glucose stimuli.

**Figure 3:**
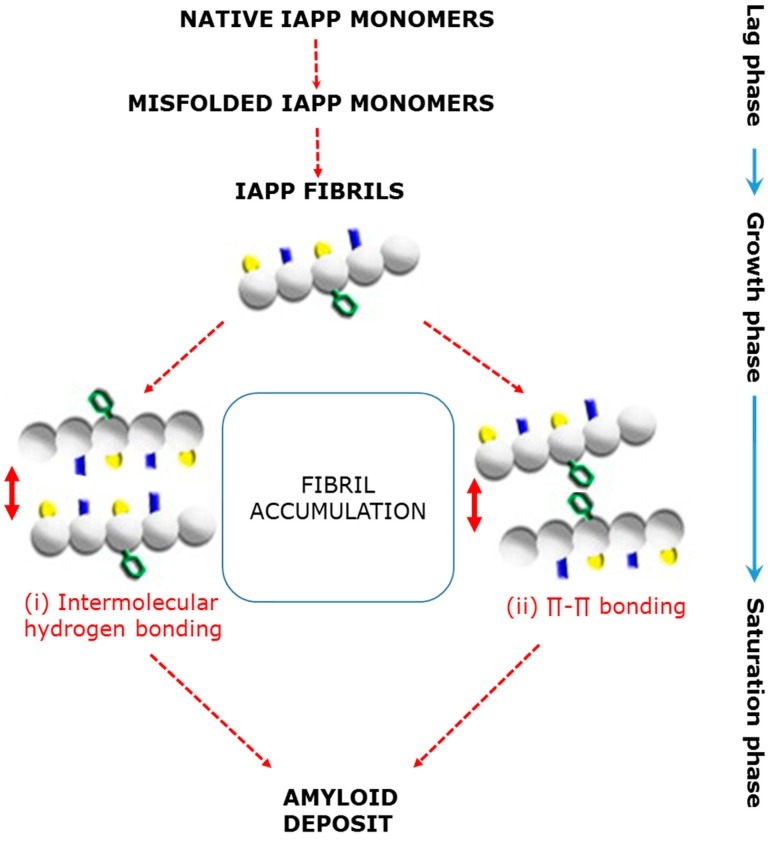
Schematic depiction of the two proposed mechanisms by which IAPP fibrils misfold and aggregate to form amyloidogenic deposits (adapted from [131]).

However, during IR and/or β-cell dysfunction, the expression of IAPP increases to that of insulin, resulting in the misfolding of pro-IAPP in the ER and/or the decreased processing of pro-IAPP in the secretory granule. The resultant misfolded and/or unprocessed pro-IAPP present in the secretory granule is released along with insulin and, extracellularly, further undergoes structural changes to initiate fibril formation. Additionally, misfolded pro-IAPP in the secretory granules may cause the contents within the granules to be targeted to the lysosome to be degraded, as the lysosomal system is responsible for the removal of excess or misfolded peptides, such as IAPP and insulin. Thus, it is also possible that fibril formation could occur intracellularly as a result of the aggregation of pro-IAPP in the lysosome, with the nascent fibrils released into the extracellular space [127]. Once these fibrils are formed either within or outside the β-cells, they provide the ‘seed/nucleus’ required to facilitate the second stage, or rapid amyloid fibril accumulation [128]. The process of further fibril formation is stabilised by intermolecular hydrogen bonding [129] to form small β-sheet cytotoxic oligomers, which eventually form the amyloid deposits. Alternatively, the protein self-assembly process involves π–π interaction, in which the aromatic residues of IAPP interact with each other via π-stacking to form the amyloid [130] (Figure 3).

A recent study of transgenic mice demonstrated that the amount of IAPP produced, along with the degree of oligomerization, differentially affects the amyloid fibril formation and its subsequent cytotoxic effects [8]. This is in line with previous work [132,133] that reported the increased cytotoxicity of oligomeric intermediates rather than the mature fibrils itself. While the exact mechanism of toxicity remains unknown, it has been suggested that the oligomers disrupt the cellular membranes, e.g., mitochondria [127], by forming pore-like structures [9], destabilising the intracellular ionic environment to generate ROS [10] and trigger apoptosis [133,134,135]. IAPP exposure in rat insulinoma, RINm5F, cells and human islet β-cells [136,137] has been shown to up-regulate pro-apoptotic genes—c-fos, fosB, c-jun, and junB [138]—in a time and concentration-dependent manner, as well as to increase the expression of apoptotic markers p53 and p21^WAFI/CIPI^ [139].

### 3.3. Targeting Amylin Misfolding and Aggregation with Polyphenol Flavonoids—An Emerging Novel Therapy for T2D

Amyloid deposits have been shown in vivo to be in a dynamic state of turnover and have the potential to regress if fibril formation is inhibited [140]. Hence, preventing or arresting the formation of amyloid-related β-cell failure at an early stage of T2D may preserve endogenous insulin secretion and prevent or delay hyperglycaemia. Two suggested mechanisms involve the inhibition of (i) the precursor pool of IAPP and (ii) the amyloid fibril. The former mechanism is postulated, given that IAPP deficiency in Type 1 diabetics (T1D) does not seem to be associated with severe clinical abnormalities. The evidence supports insulin secretion, and its subsequent effect on the rate of glucose disposal, to be lower in transgenic mice that overproduce hA than that seen in normal mice [141]. Conversely, the reverse is observed in IAPP knockout mice [142], suggesting that, in addition to exerting an anti-amyloidogenic effect, inhibiting the production of IAPP may improve glycaemic control through the inhibition of the potentially diabetogenic metabolic effects of the polypeptide. While demonstrated in vitro, the use of antisense oligonucleotides [143] or the expression of antisense complementary DNA [144] has been proposed for the direct inhibition of IAPP, to increase insulin mRNA and the protein content of cells. Again, IAPP inhibition is also proposed via an indirect mechanism, whereby a reduction in an individual’s insulin requirements will in turn reduce the production of IAPP and therefore amyloidosis. This reduction may be initiated by administering insulin therapy early in the course of T2D [145] or by the use of antidiabetic drugs, i.e., metformin.

**Figure 4:**
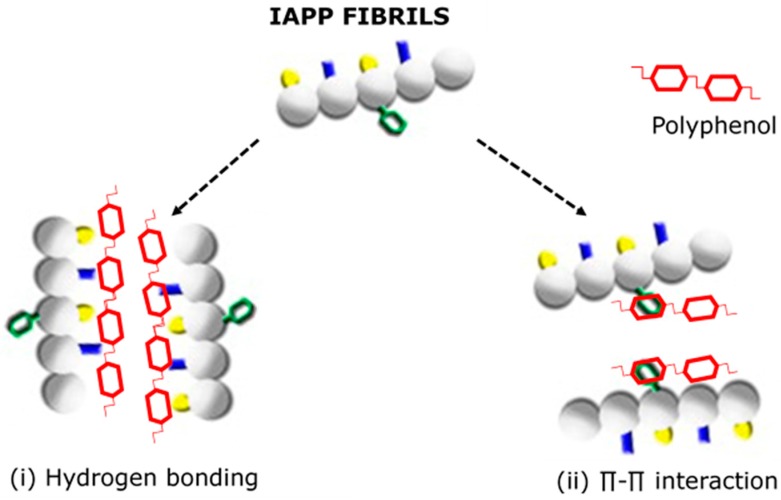
Proposed mechanisms by which polyphenols interfere with the self-assembly process of IAPP to inhibit the formation of cytotoxic oligomers (adapted from [131]).

A more attractive approach clinically is via the dissociation of amyloid fibrils during their formation in order to disrupt the β-pleated sheets and to prevent amyloidosis [146], and has been demonstrated using short synthetic peptides, containing the self-recognition motifs of the protein, engineered to destabilise the abnormal conformation to correct protein misfolding [146]. This has also been shown by binding IAPP monomers with ion ligands [147] to inhibit oligomerisation and effectively reduce amyloid cytotoxicity [148], and is thought to be the mechanism by which tetracycline exerts anti-diabetic activity [91]. A unique feature of tetracycline is that it contains an aromatic ring that facilitates the interaction with lipophilic residues of monomers (π stacking) as polar groups that can form hydrogen bonds with specific residues to strengthen the drug–protein interaction [149,150]. Likewise, polyphenols have been shown to act as small molecule inhibitors to prevent amyloid formation via similar mechanisms. According to the “π stacking” theory, the aromatic rings of polyphenols may competitively interact with aromatic residues in IAPP by sandwiching between two aromatic residues to prevent π–π interaction and block the self-assembly process [92] (Figure 4). Alternatively, the phenolic hydroxyl group of polyphenols may inhibit amyloid fibril formation by binding to the hydrophobic residues in IAPP to modulate oligomerisation [151] (Figure 4). This is similar to the mechanism by which quercetin and epigallocatechin gallate (EGCG) has also been shown to inhibit 11β-HSD1, by binding to the active site by hydrogen bond interaction [152,153].

It is important to consider that the mechanism by which polyphenols inhibit amyloid formation differs depending on which part of the assembly pathway it is involved in [154]. Accordingly, polyphenols have been shown to interact with different forms; i.e., either the monomeric, oligomeric or fibrillar forms. Some polyphenols have been shown to exert their inhibitory effects on the oligomers, while others inhibit the formation of fibrils and some others do both.

Epigallocatechin gallate (EGCG), an important polyphenol found in green tea, has been shown to redirect amyloid fibril formation from fibrillogenic forms to non fibrillogenic oligomers; i.e., “off-pathway” aggregates that are unable to form amyloid [155,156] and have been shown to protect rat insulinoma, INS-1, cells from IAPP amyloid-induced cytotoxic effects [157]. EGCG binds to the native monomers to prevent their conversion into stable, β-sheet-rich structures, which are a prerequisite for nucleation-dependent amyloid fibril assembly, thus interfering with the early stages in the amyloid formation pathway. EGCG preferentially binds to the unfolded IAPP, due to the favourable spatial distribution of the poly-hydroxyl groups on the planar aromatic rings and its ability to form covalent bonds [12,158].

Similarly, resveratrol, a main constituent of grape seeds, has been shown to bind to both monomeric and fibrillar forms and to selectively remodel soluble oligomers and fibrillary intermediates to form less toxic oligomers of IAPP [13] in pancreatic β-cell line INS-1E [159,160]. The mechanism of inhibition, however, differs from that of EGCG. Simulation studies have demonstrated that resveratrol interferes with and blocks IAPP β-sheet side chain stacking [161], especially stacking of the aromatic rings, preventing the overall aggregation of the polypeptide. Additionally, oleuropeinaglycone (OLE), works in a similar manner to EGCG and resveratrol. OLE, the main phenolic component of olive oil, has been shown to interfere with the hIAPP fibrils to prevent the formation of toxic oligomers in RIN-5 F rat insulinoma cells [162]. The compound delays the conformational transition of hIAPP and redirects it to form off-pathway aggregates that are nontoxic. OLE also modulates the cytotoxic effects of the fibrils by preventing them from permeabilising the plasma membrane.

Curcumin, the main constituent of the rhizome *C. longa*, has been extensively investigated in in vitro and in vivo studies and has been shown to inhibit the formation of fibrils of IAPP in a concentration-dependent manner [13,163,164]. While curcumin has the ability to inhibit amyloid formation, it is unlikely that it can be used to prevent amyloidogenesis at ther in approximately apeutic concentrations in T2D. This is likely as curcumin is protective in INS cells against exogenous IAPP cytotoxicity within a narrow concentration range (10–25 μM); however, it is cytotoxic when concentration was increased above 25 μM [163]. A similar effect was shown using models of endogenous overexpression of hIAPP (INS cells and h-IAPP transgenic rat islets).

Rosamarinic acid, a phenolic derivative of caffeic acid found in many *Lamiaceae* herbs, has the ability to inhibit amyloid formation and destabilise preformed IAPP amyloid [164] by specifically binding to the polypeptide to inhibit its polymerisation. Additionally, polyphenolic molecules such as ferulic acid [165], a hydroxycinnamic acid; baicalein [165], a flavonoid found in the Chinese herb *Scutellariabaicalensis*; salvionolic acid B [166], a phenolic acid found in the Chinese herb *Salvia miltiorrhiza;* and silibinin [167], an active flavonoid constituent of silymarin, have been shown to inhibit the formation of an hIAPP amyloid β-sheet, preventing the aggregation of hIAPP fibrils and suppressing toxic oligomers of hIAPP monomers to reduce islet amyloid in vitro.

Again, recent work using myricetin [168,169] has found similar inhibitory effects on IAPP amyloid formation. Kao and colleagues [170] used the extracts of 13 fruits in vitro to analyse their ability to prevent the aggregation of amyloidogenic IAPP and found that flavonols from raspberries and blueberries were the strongest inhibitors of aggregation. Of the flavonols, quercetin and quercetin-3-*O*-rutinoside (rutin) have been shown to be potent inhibitors of IAPP aggregation and share structural similarities to tetracylines [91]. It is the aromatic rings of these bioactive molecules that have been proposed to competitively interact with residues of IAPP to prevent π–π interaction in order to inhibit the self-assembly process [92]. Alternatively, it has been suggested that the hydroxyl moieties of these flavonols inhibit fibril formation by creating hydrogen bonds in the amyloidogenic protein to modulate IAPP oligomerisation. The polycyclic nature of both quercitin and rutin interact with the amyloidogenic region in hA to suppress β-sheet formation and, therefore, have been shown to promote the formation of α-helix by hA, either by allowing its spontaneous formation or by promoting its formation from random coil [93].

It is important to consider that the absorption of dietary quercetin and rutin is largely determined by the chemical structure [81] and food matrix [171], which could limit digestion and metabolism [53], likely resulting in a lower bioavailability when compared to vitamin antioxidants [172]. Additionally, intestinal permeability [173] or co-administration with ascorbic acid [174] present in foods may influence the kinetics of absorption. Partial absorption occurs in the stomach and small intestine, the latter via hydrolysis by two different routes through the action of endogenous β-glucosidases—lactase phloridzin hydrolase (LPH) and cytosolic-β-glucosidase (CBG)—to generate more lipophilic, and thereby absorbable, aglycones [175]. While LPH is expressed at the brush border of enterocytes to selectively absorb quercetin glucoside [176], CBG hydrolyses conjugated glycosides that have been previously transported within enterocytes via the active sodium-dependent glucose transporter (SGLT1) [176,177,178]. Rutin, on the other hand, is not absorbed in the small intestine [179] and requires metabolism by colonic microflora [180]. Accordingly, in a comparative study, it was reported that quercetin glycosides are mostly absorbed in the stomach and small intestine, reaching peak serum concentration (C_max_) in approximately 1.5 h, while absorption of rutin, which is dependent on the release of aglycones by the large bowel microbiota, reaches C_max_ later, in approximately 5.5 h [81]. These segmental differences in absorption are in line with reports by Hollman et al. [181,182,183], who showed that maximal absorption occurs at 0.5–0.7 h and 6–9 h following the ingestion of quercetin-4′-glucoside and rutin, respectively; hence the relatively low bioavailability of rutin (20%) compared to that of the glucoside moiety. Glycoside moiety is therefore a major determinant of the absorption of flavonoids to define biological activity and, in part, explains the varied responses from dietary intakes [184]. It is noteworthy that, prior to reaching portal circulation, these flavonoids can undergo phase II metabolism by methylation, sulfation and glucuronidation [56]. It is therefore pertinent to consider the levels of dietary intake to maintain physiological concentrations for novel mechanisms to be operational, which are difficult to achieve in plasma and extracellular fluids, with flavonoid concentrations reported in the micromolar range in these biological compartments [53]. Accordingly, intracellular levels have been estimated to reach picomolar or nanomolar concentrations [185]. Several approaches, including ingestion along with dietary fat [186,187], to promote the rafficking of quercetin and rutin across the gastrointestinal mucosa to increase bioaccumulation have been advocated [173,188]. Ergo, supplementation with 500 mg rutin once daily for six weeks, a concentration equivalent to that used to ameliorate IAPP amyloid in human amylin transgenic (hAtg) mice [93], has been shown to increase circulating rutin or quercetin levels by at least 2.5 fold [189].

In vivo studies support the use of quercetin [94] and rutin [93] as antioxidants that additionally modulate IAPP aggregation, the latter using transgenic mice engineered to develop features of T2D in humans. This is in line with other studies that have shown that rutin interacts with soluble hA oligomers to prevent the formation of cytotoxic aggregates and to protect against Fas-mediated destruction of islet cells [8,91]. hAtg mice with β-cell specific IAPP expression [8] replicate the T2D phenotype of islet changes to develop diabetes [190], with rutin treatment shown to prolong the onset of diabetes and ameliorate the severity of diabetic syndrome in treated mice [93]. Hence, studies utilising human amylin transgenic (hAtg) models provide reliable critical mechanistic information [91,126,137] contributing toward our understanding of IAPP oligomerisation, its related cytotoxicity and resultant islet cell apoptosis. However, the beneficial effects of quercetin and rutin have not been evident in other studies [158]. Notably, it has been suggested that the rutinoside group, following ingestion of rutin, may be cleaved during intestinal transit to release quercetin and its glycosides, which could act as the bioactive compounds rather than rutin itself. However, this was disproved in a pharmacokinetic study that did not find free circulating quercetin in blood following dosage with rutin and quercetin-4-*O*-glucoside [191], possibly due to the degradation of rutin by colonic microflora into phenolic catabolites: 3,4-dihydroxyphenylacetic acid, 3-methoxy-4-hydroxyphenylacetic acid and 3-hydroxyphenylacetic acid [192]. Again, the simultaneous detection of quercetin and rutin in plasma as well as lymph, following intra-duodenal administration in rats, seems to indicate that rutin may possibly be absorbed intact from intestinal cells [193]. This finding was in line with previous publications conducted in animal models [194,195]. While validation from human studies is certainly required, plasma quercetin has often been measured as a marker of rutin absorption to facilitate the ease of analyses [189]. While both the relative and absolute bioavailability of quercetin has been assessed, studies examining the former have most commonly been used in human studies.

## 4. Learnings from the Evidence and Concluding Remarks

T2D prevalence and its associated micro and macro-vascular risks continue to rise as a result of over-nutrition and lifestyle changes [196]. Accordingly, safer, natural and well-tolerated compounds, such as polyphenols that are widely available from dietary sources, with established antidiabetic effects, are emerging as novel therapeutic targets for delaying the progression and for preventing T2D in ‘at-risk’ individuals [63]. In addition to being anti-oxidant and anti-inflammatory agents [197], polyphenols exhibit anti-hyperglycaemic effects, as they improve carbohydrate metabolism, β-cell function and insulin resistance. This includes its novel role in arresting IAPP fibril formation and inhibiting the deleterious cytotoxic effects of IAPP amyloid.

Loss of functional β-cell mass is central to the pathophysiology of T2D [6]. IAPP misfolding and aggregation has recently emerged as a critical entity in islet cell pathology and T2D progression, with unequivocal data suggesting that the inhibition of the cytotoxic IAPP oligomers is key to improving pathology [97]. Whether these aggregates are a consequence of the tissue damage during disease progression or involved in disease pathogenesis remains to be determined. It is likely the latter, given that PMD is an established mechanism in the pathogenesis linked to the various neurodegenerative diseases [99]. On consideration, IAPP misfolding may play an important role in the transition from the prediabetic state to that of T2D and warrants further investigation.

Using polyphenol flavonoids to target amyloid deposits appears to be a rational, promising and novel therapeutic approach, given that the favourable phenolic ring structure and hydroxyl moieties allows them to function as potent inhibitors of IAPP oligomerisation. The benefits of using flavonoid polyphenols is that they occur naturally, have antioxidant properties, are stable in biological fluids, have the ability to cross the blood brain barrier and do not elicit an immune response. In addition, they have also been shown to inhibit 11β-HSD1 [152,153,198] and lower cortisol secretion. Dysregulated cortisol secretion has been associated with the risk of T2D [199] and increased complications [200,201], especially greater diurnal secretion [202].

It is noteworthy that, although anti-oxidative properties of polyphenol flavonoids have been associated with the reduced risk of T2D, further research to elucidate the mechanism are required. ROS activity in in vitro studies has been observed at concentrations that are significantly higher than the physiological levels found in vivo [189]. Hence, while in vitro studies show positive effects, short term intervention studies are required to address and further elucidate the effects of these compounds in individuals diagnosed with prediabetes and T2D to address these proposed mechanisms of action. Discretion on the type and class of polyphenol, along with dosage, will be an important determinant of the outcomes, given the differences in bioavailability following dietary intake. Of the polyphenol flavonoids, the flavonol quercetin [90], the most abundantly present in the studied diet, and its rutinoside conjugate, rutin [93,203], may likely be most relevant to ameliorating the T2D risk, given that they are potent antioxidants [188], attenuate fasting and postprandial hyperglycaemia [204,205], and been shown to strongly inhibit IAPP-induced cytotoxicity.

## Figures and Tables

**Table 1:** Dietary quercetin and rutin-content in common foods.

Food Source	Quercetin (mg/100 g)	Rutin (mg/100 g)
Apple (with skin) *	3.80	0.22
Broccoli (raw)	2.25	1.6
Buckwheat groats (raw)	3.47	23.0
Grape skin (red)	1.05	149.1
Raspberry (red)	1.10	11.0
Cocoa powder (unsweetened)	10.0	-
Onion (raw)	20.30	0.68
Spinach (raw)	3.97	-
Black tea (brewed) **	2.19	1.62
Green tea (brewed)	2.49	1.46
Fruit tea (pomegranate)	0.00	632
Red wine ***	2.11	0.81

Data obtained from the United States Department of Agriculture and is determined by column or high-performance liquid chromatography, capillary zone electrophoresis, or micellar electrokinetic capillary chromatography [75,76,77,78,79,80]. * Apples reported as Gala apples. ** Brewed Tea (mg/100 g (100 mL)): tea infusions equivalent to 1 g of dry tea. Infusion values are standardised to 1% infusion (1 g tea leaves/100 mL boiling water) *** Red wine reported as Syrah or Shiraz.
